# Post Cryptococcal Moyamoya Syndrome in Adult Human Immunodeficiency Virus Patient With Anterior and Posterior Circulation Involvement: Case Report

**DOI:** 10.7759/cureus.44052

**Published:** 2023-08-24

**Authors:** Kidist T Arficho, Cezar Gumma, Mathew N Chakko

**Affiliations:** 1 Radiology, Ascension Providence Hospital, Michigan State University College of Human Medicine (MSUCHM), Southfield, USA; 2 Radiology, Beaumont Hospital, Farmington Hills, USA; 3 Neuroradiology, Ascension Providence Hospital, Michigan State University College of Human Medicine (MSUCHM), Southfield, USA

**Keywords:** human immunodeficiency virus (hiv) infection, hiv/aids related opportunistic infections, computed tomographic angio graphy, moyamoya disease (mmd), hiv aids, pathogenesis of moyamoya disease, moyamoya angiopathy, cryptococcal meningitis, moyamoya syndrome

## Abstract

Moyamoya disease (MMD) is a rare idiopathic progressive vaso-occlusive disease characterized by irreversible vascular occlusion and collateral development of distal internal carotid arteries. Initially perceived as an exclusive entity to the East Asian population, the disease is now being reported globally, affecting individuals of diverse ethnicities. We present a case of a 55-year-old African American male patient with human immunodeficiency virus/acquired immunodeficiency syndrome (HIV/AIDS) and a prior history of cryptococcal meningitis presenting to the emergency department with recurrent episodic headaches, which was refractory to routine medical therapy. Neuroimaging with computed tomography angiogram of the head and neck and magnetic resonance imaging of the brain led to the subsequent diagnosis of moyamoya syndrome (MMS). To our knowledge, MMS is uncommon in adult HIV/AIDS patients. It is crucial that clinicians are aware of the disease progression. For effective recognition and prevention of the condition, it is of utmost importance that clinicians possess a comprehensive understanding of the disease and its clinical manifestations.

## Introduction

Moyamoya disease (MMD) is a gradual narrowing and blockage of the terminal portions of the bilateral internal carotid arteries and their proximal branches, known as steno-occlusive vasculopathy. As a result of this occlusion, collateral vessels develop to compensate for the arterial occlusion [1,2]. Moyamoya syndrome (MMS) refers to moyamoya angiopathy associated with underlying conditions like infection. While it is more commonly observed in children and young adults, its occurrence in older adults, particularly in the context of human immunodeficiency virus (HIV) infection, is uncommon [2]. We present a unique case of MMS and its temporal relationship with cryptococcal meningitis in a patient with HIV infection. 

## Case presentation

A 55-year-old man with a past medical history of HIV/acquired immunodeficiency syndrome (AIDS) was admitted to the emergency department in 2017 for an intractable headache. The CT and MRI of the brain showed chronic lacunar infarct in the basal ganglia without acute abnormality. CT imaging performed at a separate emergency visit for the reported headache was unremarkable. 

**Figure 1:**
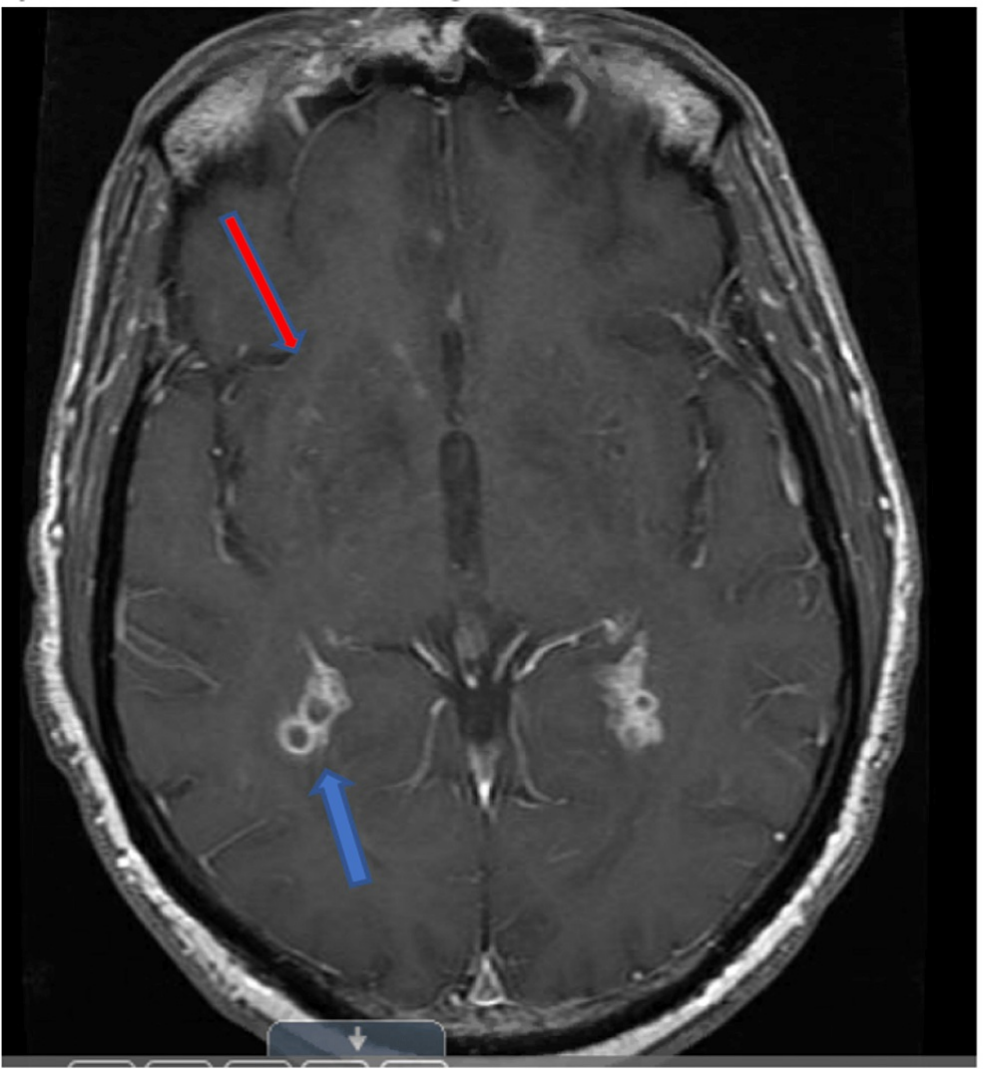
Contrast-enhanced magnetic resonance imaging shows perivascular enhancement in right basal ganglia (red arrow) and prominent enhancement of choroid plexus (blue arrow).

**Figure 2:**
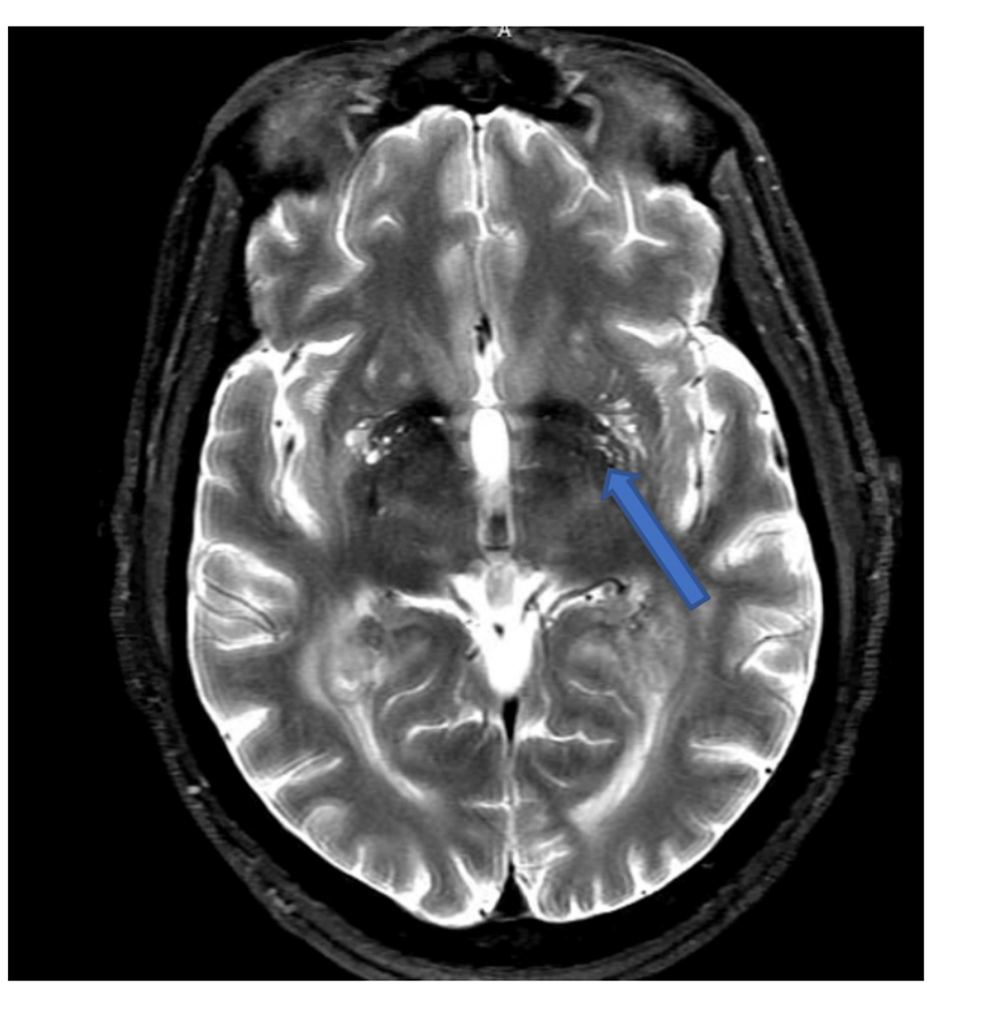
Dilated Virchow-Robin spaces with multiple cystic lesions shown at the bilateral basal ganglia on T2WI (blue arrow).

A year after his initial presentation, the patient presented to the emergency department with a headache. The brain MRI showed nodular enhancement of the ventricular ependyma along the anterior inferior surface of the lateral ventricles, which extended from the frontal horns to the trigones and temporal horns. On post-contrast scans, choroid plexuses within the atria and occipital horns were enlarged and heterogeneously enhanced. Bilateral foci of clustered ring enhancements were most pronounced in the trigones of the lateral ventricles (Figure 1). Interval development of prominence of the bilateral Virchow-Robin spaces in the basal ganglia with mild enhancement was seen (Figure 2). Based on a brain MRI, history of HIV, positive cerebrospinal fluid cryptococcal antigen, and culture, cryptococcal meningitis and choroid plexitis were diagnosed.

**Figure 3:**
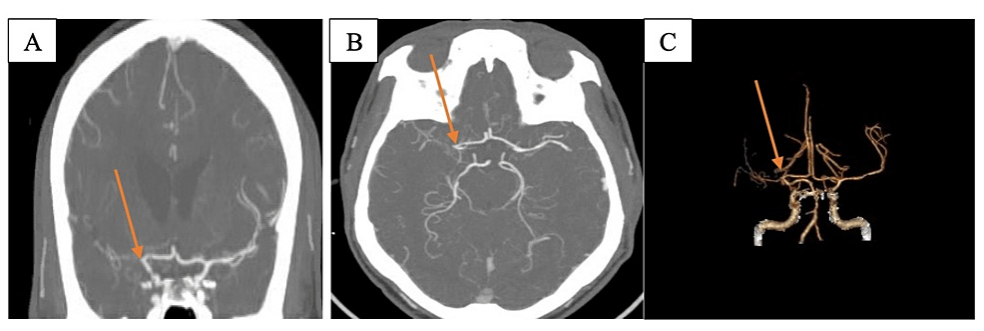
(A-C) CT angiography revealed marked attenuation of the terminus of the right supraclinoid internal carotid artery and near occlusion of the right middle cerebral artery (arrows).

Four years later, the patient presented to the emergency department again with a complaint of headache. CT angiography revealed marked attenuation of the terminus of the right supraclinoid internal carotid artery and near occlusion of the right middle cerebral artery (Figure 3).

**Figure 4:**
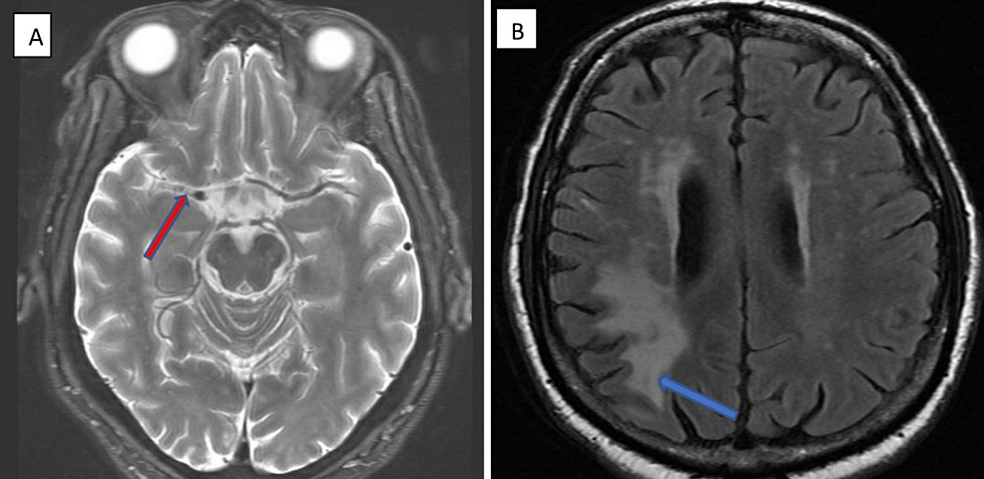
(A) T2 weighted axial MRI shows an absence of signal void in the right middle cerebral artery (red arrow). (B) T2 fluid-attenuated inversion recovery image shows chronic infarct with encephalomalacia involving the right posterior parietal lobe (blue arrow).

**Figure 5:**
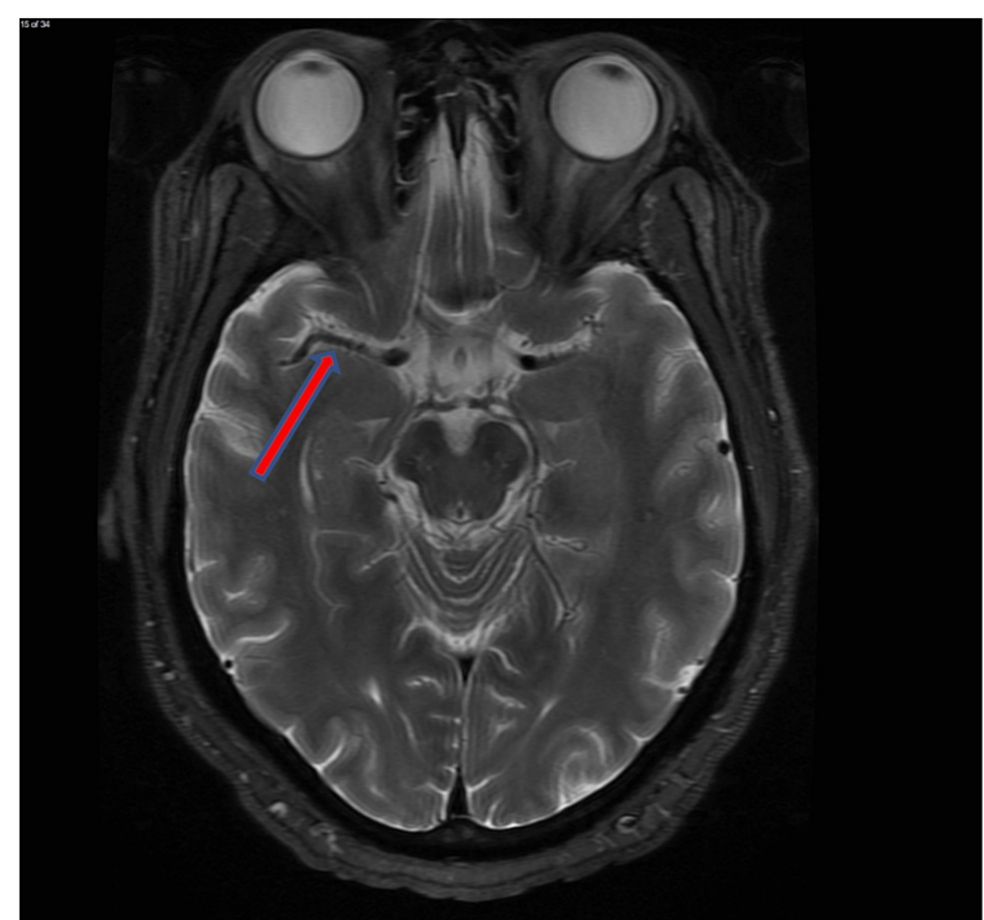
T2 weighted MRI from 2 years earlier shows patent right middle cerebral artery (red arrow).

Brain MRI demonstrated diminished flow void of the right middle cerebral artery, representing the vascular occlusion and collateral formation (Figure 4A). T2 fluid-attenuated inversion recovery image of old infarct in the right posterior parietal lobe (Figure 4B). Patent flow voids were visible on the prior MRI from two years earlier which suggests vascular occlusion occurs only after the initial course of cryptococcal meningitis (Figure 5).

**Figure 6:**
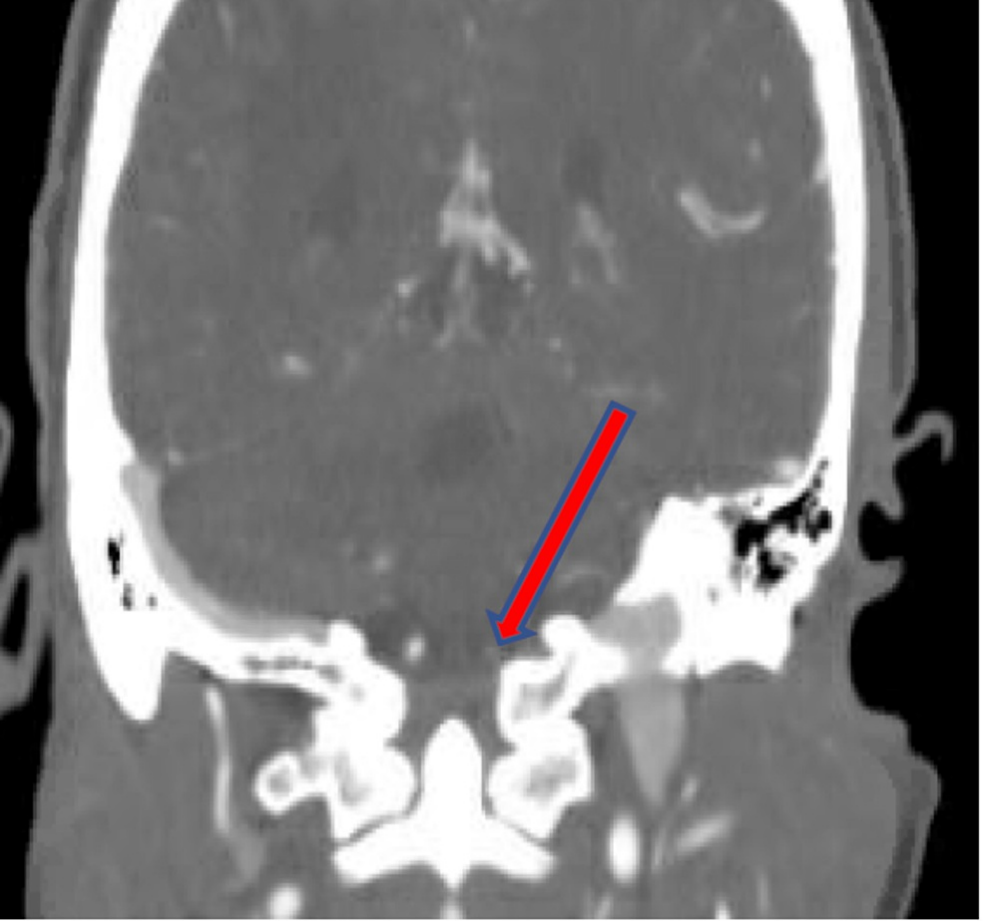
Coronal view of CT angiography shows attenuation to near occlusion of the left V4 segment of vertebral artery (red arrow).

**Figure 7:**
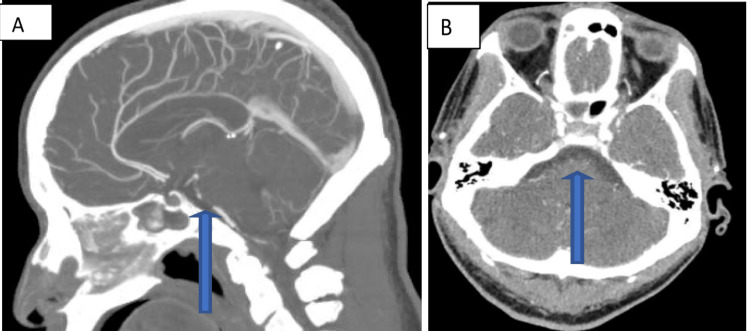
(A and B) Sagittal and axial views of CT angiography show short-segment complete occlusion of the proximal basilar artery with distal reconstitution (blue arrows).

The most recent CT angiography five years later showed attenuation to near occlusion of the left V4 segment of the vertebral artery (Figure 6) and complete occlusion of the proximal basilar artery with reconstitution, and severe attenuation distally (Figure 7).

## Discussion

MMD is a chronic cerebrovascular condition of unknown cause, marked by the narrowing and blockage of the intracranial blood vessels. This condition is accompanied by the formation of new irregular collateral blood vessels as a compensatory response. In most cases, moyamoya disease appears bilaterally. However, moyamoya may initially occur unilaterally and proceed gradually into bilateral changes [1,2]. According to the description by Suzuki et al., moyamoya disease involves the constriction and reduction in the diameter of the internal carotid arteries, and it is identified by the existence of atypical, mesh-like collateral blood vessels observed on cerebral angiograms. The term "moyamoya", has its roots in the Japanese language and refers to a hazy and indistinct phenomenon, similar to the appearance of a “puff of cigarette smoke” [2]. 

Moyamoya disease primarily affects the anterior circulation in the brain. However, the disease affects the posterior circulation in about 30% of the cases [3-5]. The most frequent associations are head and neck irradiation, previous meningitis, cerebral vasculitis, autoimmune disease, prothrombotic disorders, hematologic diseases, genetic/chromosomal disorders, drugs like oral contraceptives, neoplasms, and cranial trauma6. The disease commonly manifests as cerebrovascular episodes, which can be either ischemic or hemorrhagic, accompanied by various neurological presentations with notable differences in clinical features between children and adults; most children experience a transient ischemic attack or cerebral infarction, while adult patients may develop intracranial hemorrhage, cerebral infarction or a combination of these [1-4]. Moyamoya disease shows its highest incidence peaks in two specific age groups: young children around the age of five and middle-aged adults in their mid-40s [5-8].

Generally, MMD was commonly linked to Asian people, but currently, it has been recognized to occur in individuals from various ethnicities around the World, including European and North American populations. MMD predominantly impacts individuals of Asian descent, especially Japanese, Korean, Chinese, and Indians, and the incidence is approximately 0.54/100,000/year, with a prevalence of 6/100,000 [7]. In the United States, the annual incidence rate of MMD/MMS is 0.086 per 100,000 persons, about 10 times lower than in East Asian countries [3]. MMD is rarely seen in the African American population; Uchino’s study highlights that the State of California and Washington reported 27 cases of MMD among African American descent. Moreover, based on the 2000 US Census population data, the annual diagnosis of moyamoya in the African community was limited to only 44 cases [3]. Our patient is African American with no known prior medical, surgical, or treatment history and was diagnosed with HIV and cryptococcal meningitis in 2017. These findings emphasize the need for greater awareness and understanding of the disease’s prevalence and impact across different ethnic groups.

Imaging plays a crucial role in the diagnosis of moyamoya vasculopathy. It is characterized by stenosis of the distal internal carotid arteries (ICAs) along with their proximal branches. The presence of moyamoya vessels and collateral circulation indicates neovascularization. Posterior vascular involvement is seen in one-third of cases and is likely an extension of the stenotic process from the anterior circulation. The occlusive lesions in the posterior circulation show steady progression like the pattern seen in the anterior circulation. Occlusion of ICAs, basilar and vertebral arteries exist in our case, which may have the same etiology [9].

Determining the actual occurrence of cerebrovascular complications in AIDS is challenging to establish due to the frequent association between HIV and opportunistic infections. Morgello et al. previously described the association between moyamoya and HIV/AIDS in adults. They documented a rare case of quaternary neurosyphilis in a 22-year-old Haitian man, which resulted in MMS [9]. Our case is an African American male patient with no known previous medical illness who was diagnosed with HIV/AIDS and cryptococcal meningitis before the diagnosis of moyamoya angiopathy was made. 

The mechanisms underlying this association are not well understood. It has been suggested that HIV infection or complications of opportunistic infections result in postinfectious central nervous system (CNS) vasculitis. The pathogenesis of CNS vasculitis in HIV/AIDS involves various mechanisms, such as infection of endothelial cells by HIV or other opportunistic infections, immune complex deposition and dysregulation of cytokines, and attaching syntax. Alterations of the vascular systems and vasospasm result in ischemia and thrombosis leading to arterial narrowing of the supraclinoid portion of the internal carotid arteries with vessel wall irregularities, focal dilations, and thrombo-occlusions [9-13]. However, delayed postinfectious vasculitis is less frequently reported [14-19]. The association between post cryptococcal meningitis and MMS was described by Maramattom in an immunocompromised patient who had undergone live-related donor kidney transplantation. She had been continuing triple immunosuppression with oral prednisolone, tacrolimus, and mycophenolate [15]. A comprehensive understanding of these diverse mechanisms is crucial in devising effective management and treatment approaches for individuals with CNS vasculitis in the context of HIV/AIDS. Our case report highlights the potential relationship between these conditions and emphasizes the importance of further investigation in understanding their interplay and implications for patient management.

## Conclusions

In summary, further research is required to explore the pathogenesis of moyamoya syndrome in the context of HIV infection. It is plausible that opportunistic infections linked to HIV might be associated with the pathological changes that give rise to cerebrovascular disease and the development of the moyamoya phenomenon. Gaining a deeper understanding of these connections can offer valuable insight into the complex interplay between HIV and MMS, potentially leading to advancements in diagnosis and treatment strategies for affected individuals. We describe a case of MMS in an HIV patient who presented several years after cryptococcal meningitis. There could be an association between cryptococcal meningitis and the later development of MMS. This case highlights uncommon involvement of the anterior and progressive involvement of the posterior artery system, which is considered as the whole cerebral artery-involved type MMS. Furthermore, our case report emphasizes the infrequent occurrence of moyamoya syndrome in the African-American population and highlights the significance of employing CT angiography as a valuable diagnostic modality for its detection. To our knowledge, there is no reported case of MMS in an HIV patient with a history of post cryptococcal meningitis and subsequent occlusion of the whole cerebral artery. Awareness of the presentation and evolution of MMS in HIV patients is important to permit early intervention and improve outcomes. 
